# Stabilization of RDT target antigens present in dried *Plasmodium falciparum*-infected samples for validating malaria rapid diagnostic tests at the point of care

**DOI:** 10.1186/s12936-017-2155-7

**Published:** 2018-01-08

**Authors:** Collins Morang’a, Cyrus Ayieko, George Awinda, Rachel Achilla, Caroline Moseti, Bernhards Ogutu, John Waitumbi, Elizabeth Wanja

**Affiliations:** 1 Maseno University, P.O Box Private Bag, Maseno, Kenya; 2 United States Army Medical Research Directorate, P.O Box 54, Kisumu, 40100 Kenya; 3 Kenya Medical Research Institute, P.O. Box 54840-00200, Nairobi, Kenya; 4 United States Army Medical Research Directorate-Armed Forces Research Institute of Medical Sciences, Bangkok, 10400 Thailand

**Keywords:** Malaria, Rapid diagnostic tests, Chemical additives, Stability, Positive controls

## Abstract

**Background:**

Malaria rapid diagnostic tests (RDTs) are a great achievement in implementation of parasite based diagnosis as recommended by World Health Organization. A major drawback of RDTs is lack of positive controls to validate different batches/lots at the point of care. Dried *Plasmodium falciparum*-infected samples with the RDT target antigens have been suggested as possible positive control but their utility in resource limited settings is hampered by rapid loss of activity over time.

**Methods:**

This study evaluated the effectiveness of chemical additives to improve long term storage stability of RDT target antigens (HRP2, pLDH and aldolase) in dried *P. falciparum*-infected samples using parasitized whole blood and culture samples. Samples were treated with ten selected chemical additives mainly sucrose, trehalose, LDH stabilizer and their combinations. After baseline activity was established, the samples were air dried in bio-safety cabinet and stored at room temperatures (~ 25 °C). Testing of the stabilized samples using SD Bioline, BinaxNOW, CareStart, and First Response was done at intervals for 53 weeks.

**Results:**

Stability of HRP2 at ambient temperature was reported at 21–24 weeks while that of PAN antigens (pLDH and aldolase) was 2–18 weeks of storage at all parasite densities. The ten chemical additives increased the percentage stability of HRP2 and PAN antigens. Sucrose alone and its combinations with Alsever’s solution or biostab significantly increased stability of HRP2 by 56% at 2000 p/µL (p < 0.001). Trehalose and its combinations with biostab, sucrose or glycerol significantly increased stability of HRP2 by 57% (p < 0.001). Unlike sucrose, the stability of the HRP2 was significantly retained by trehalose at lower concentrations (500, and 200 p/µL). Trehalose in combination biostab stabilizer increased the percentage stability of PAN antigens by 42, and 32% at 2000 and 500 p/µL respectively (p < 0.01). This was also the chemical combination with the shortest reconstitution time (~ < 20 min).

**Conclusions:**

These findings confirm that stabilizing RDT target antigens in dried *P. falciparum*-infected samples using chemical additives provides field-stable positive controls for malaria RDTs.

**Electronic supplementary material:**

The online version of this article (10.1186/s12936-017-2155-7) contains supplementary material, which is available to authorized users.

## Background

Malaria is still a leading cause of morbidity and mortality, with 212 million cases and 429,000 deaths reported in 2016 [1]. Rapid diagnostic tests (RDTs) are a major milestone in point-of-care malaria diagnosis in an effort to attain universal access to parasite based diagnosis, consistent with WHO strategies and recommendations [2]. RDTs have several advantages such as ease of use, inexpensive, and real-time diagnosis; but field assessment studies have shown that they can report false results [3]. During transportation and storage in tropical climates the quality of RDTs deteriorates due to high temperatures and humidity [4]. Albertini et al. showed that during transport and storage of health commodities, temperatures and humidity can exceed 30 °C and 94%, respectively in Burkina Faso, Senegal, Ethiopia, and the Philippines [5]. The WHO has set out measures to ensure good manufacturing quality, evaluation programmes for lot testing (pre and post purchase), and provides guidelines for selection of RDTs before purchase [6]. But, lack of proper quality control and quality assurance mechanisms for malaria RDT during transportation and storage remains a major problem [7]. Therefore, there is need to develop positive controls for evaluating the quality of RDTs upon transportation and local storage in health facilities.

Positive control wells (PCW) based on recombinant malaria antigens are the most feasible approach to ensuring quality control of RDT in field operational use [8]. Despite the prospective evaluation of the prototype in a malaria endemic area; they still face challenges such as; validation, long-term storage stability, and technical specifications which are still under development [8]. While the world waits for the complete development of PCW, dried *Plasmodium falciparum*-infected samples were developed as possible cheaper alternatives in monitoring the performance of RDT in routine use [9]. The dried *P. falciparum*-infected sample tubes contain the RDT target antigens; histidine rich protein 2 (HRP2), lactate dehydrogenase (LDH) and aldolase [9, 10]. The challenge facing their applicability is protein degradation and rapid loss of reactivity during prolonged storage at ambient conditions [9, 10]. Furthermore, it is challenging to establish well characterized samples for specific RDTs, as well as quantification of target antigens. Consequently, there is need to stabilize these target proteins to ensure their conformational stability during transport and storage in typical field conditions.

Protein stabilization involves the prevention of the irreversible loss of the unique chemical structure of the protein [11]. Some of the methods used to stabilize proteins for long-term storage include; ligand stabilization, storage as frozen solutions, salted out precipitates, freeze dried solids, and chemical additive stabilization [12]. Chemical additives prevent the loss of enzymatic activity, prevent denaturation, inhibit irreversible aggregation, and protect proteins against chemical instabilities [13]. The commonly used chemical additives are; salts such as sodium and potassium; as well as organic solvents such as polyethylene glycol, polyethyleneimine and glycerol; and sugars such as trehalose, sorbitol and sucrose [14].

Trehalose stabilizes proteins by various mechanisms, such as vitrification, preferential exclusion, water replacement, hydrogen bonding, and non-specific thermodynamic stabilization [15]. Sucrose stabilizes proteins majorly by preferential exclusion; whereby it increases the chemical potential of the protein which adopts a more stable conformation [16]. Glycerol has also been shown in previous studies to stabilize proteins by interacting preferentially with the hydrophobic parts of the protein structures [17]. Alsever’s solution and LDH stabilizer are commercially developed for stabilizing proteins during long term storage [18, 19]. Biostab enzyme stabilizer has been shown previously to stabilize lysozyme over storage time by preferential hydration on the surface of the protein [20].

This study hypothesized that addition of commercially available chemical additives to dried *P. falciparum*-infected samples may potentially improve the long-term storage stability of HRP2, pLDH and aldolase. Therefore, this study aimed to investigate the effect of ten chemical additives on the stability of HRP2, pLDH and aldolase in during 53 weeks of storage.

## Methods

### Study design and sample collection

The study was conducted at the Walter Reed Project, Kisumu a malaria holo-endemic area where transmission occurs throughout the year [21]. The study utilized archived whole blood samples collected from the Kombewa Clinical Research Center under a WRAIR/KEMRI approved protocol #1720. Cultured samples were obtained from cryopreserved stocks of *P. falciparum* strains (3D7) maintained at the Walter Reed Project-Kisumu and used to initiate a continuous culture according to the WRAIR/KEMRI approved protocol #1919. All the collected samples were characterized by expert microscopy and diluted to three experimental parasite densities i.e. 2000, 500, and 200 p/µL using malaria negative group O+ blood. The Group O+ blood was obtained through a USAMRD-K approved study (Scientific Steering Committee; SSC#1919).

### Selection of malaria rapid diagnostic tests (RDT)

The RDTs were selected based on performance in Round 2 of WHO/FIND/CDC malaria RDT performance evaluations, national guidelines on required performance of RDTs in Kenya, and ability to detect two parasite antigens (WHO/FIND/CDC, 2010). Four different types of RDTs were selected based on panel detection score (PDS) at 200 parasite/μL of ≥ 90 (Table 1).

**Table 1:** Selected malaria rapid diagnostic tests (RDTs)

Product	Number of bands	Parasite specificity	Manufacturer	Product code	*Pf* PDS (200 parasites/μL)
CareStart Malaria HRP2 (Pf)	2	*Pf*	Access Bio, Inc.	G0141	99
First Response Malaria Ag Combo (PLDH/HRP2)	3	*Pf* and Pan	Premier Medical Corporation Ltd	II6FRC30	100
SD BIOLINE Malaria Ag Pf/Pan (HRP2/pLDH)	3	*Pf* and Pan	Standard Diagnostics, Inc.	05FK60-02-3	96
BinaxNOW Malaria Ag pf/Pan (HRP2/Aldolase	3	*Pf* and Pan	Binax, Inc., Inverness Medical, ME, USA	665-025	99

*Pf*, *Plasmodium falciparum*; PDS, parasite detection score; Pan, pLDH or aldolase antigens

### Selection of chemical additives

The selection was based on the literature review on the ability of the chemicals to improve stability of proteins. Additives chosen for this project were polyethylene glycol (Sigma-Aldrich, MO, USA), sucrose (Sigma-Aldrich, MO, USA), biostab enzyme stabilizer (Sigma-Aldrich, MO, USA), Alsever’s Solution (Fisher Scientific, USA), trehalose (Fisher-Scientific, USA), lactate dehydrogenase stabilizer solution (The Gwent Group UK), polyethyleneimine (Fisher-Scientific, USA), and glycerol (Sigma-Aldrich, MO, USA). Stepwise exploratory tests (36 tests) were conducted to determine which additives, combinations and concentrations gave optimal RDT test outcomes. The tests were conducted by adding the additives to start-up culture samples individually (mono) or in combination (mixed) with other additives at concentrations of 200 and 2000 p/µL.

The ratio of additives used in combination was 1:1 (additive: additive) as in previous studies [22]. The ratio of additives or combinations to culture was 1:2 (additive: blood), so as to achieve appropriate concentration of the additive in the blood [23]. A control start-up sample (no additive/s added) was also included. In the event that an additive, combination or concentration was found to interfere with RDT test results, that particular additive or combination was withdrawn. Additives that showed no or little effect on the stability of the proteins were also withdrawn based on weekly retesting results of the samples during 4 weeks of storage on SD Bioline and First Response Malaria Kit. Ten additives/combinations were selected from the 36 combinations (Table 2). The concentration formulations were selected according to extensive previous studies on the effect of different additives on keeping the structure of proteins [14, 24, 25].

**Table 2:** Selected chemical additives for stabilization of malaria RDT target antigens

Product name	Abbreviation	Concentration used	Manufacturer
Sucrose	Suc	0.5 M	Sigma-Aldrich, MO, USA
Glycerol/sucrose	Gly/Suc	0.5 M and 10%	Sigma-Aldrich, MO, USA
Alsever’s/sucrose	Als/Suc	100% w/v and 0.5 M	Fisher Scientific, USA & Sigma-Aldrich, MO, USA
Trehalose	Treh	0.5 M	Fisher-Scientific, USA
Sucrose/trehalose	Suc/Treh	0.5 and 0.5 M	Fisher-Scientific, USA & Sigma-Aldrich, MO, USA
Glycerol/Trehalose	Gly/Treh	10% and 0.5 M	Fisher-Scientific, USA & Sigma-Aldrich, MO, USA
Trehalose/biostab	Treh/Bio	0.5 M and 5% w/v	Fisher-Scientific, USA
Biostab/sucrose	Bio/Suc	5% w/v and 0.5 M	Sigma-Aldrich, MO, USA
LDH stabilizer	LDH Stab	100% W/v	The Gwent Group, UK
LDH stabilizer/trehalose	LDH Stab/Treh	100% w/v and 0.5 M	Fisher-Scientific, USA & The Gwent Group, UK

LDH, Lactose dehydrogenase; M, Molar; % W/v, percentage weight/volume

### Stabilization of *Plasmodium* proteins

The ten selected additives and their combinations were added to the patient and culture samples (after characterization to respective parasite densities) in a ratio of 1:2, and a control patient and culture sample (without any additive) were also prepared. Baseline data was collected by testing of all the samples to confirm the reactivity of the antigens using SD Bioline, CareStart, First Response and BinaxNOW. The test was conducted according to manufacturer instructions. Reactivity or presence of the HRP2, PLDH, and aldolase was demonstrated by color changes at the test lines. A successful test was confirmed by the presence of a control line. Results were captured as either positive or negative through visual examination of the kit. It was recorded positive if both the control band and the tests bands were visible and it was recorded negative if the control line was visible, but the test lines were absent.

Dried *P. falciparum*-infected samples in tubes were prepared by depositing 40 µL aliquots at the bottom of 2 mL vials, the tubes were left uncapped to air-dry overnight in a bio-safety cabinet, and then sealed tightly by closing the vial cap. The tubes containing dried *P. falciparum*-infected sample (stabilized and controls) were stored at room temperature. The temperature of the room was monitored daily, and recorded three times in a day on temperature charts. The average temperature during the study period was 25 °C (range 23–27 °C). Temporal stability data was collected by retesting the dried *P. falciparum*-infected samples consecutively after 1, 4, 8, 12, 15, 18, 21, 24, 33, 43 and 53 weeks of storage. The study selected 53 weeks of evaluation because, in developing countries like Kenya, there are existing trends of laboratory stock outs in peripheral health facilities due to poor infrastructure and procurement processes [26]. Therefore, stability of antigens for up-to 53 weeks will reduce the chances of facilities having stock-outs, reduce associated costs of resupply, and ensure reliability in using the positive controls.

Retesting was completed by rehydrating the dried blood pellets for the control samples and each additive stabilized sample using phosphate buffered saline with Tween-20. The tubes were left for 1 h to ensure complete rehydration. Each dried *P. falciparum*-infected sample was tested on SD Bioline, CareStart, First Response and BinaxNOW. This study defined loss of reactivity as the number of weeks after which a sample tested negative (complete loss of reactivity; 4 + to 0) on a rapid diagnostic test. Potential degradation of RDTs was done on the batch, if a sample was recorded negative (lost reactivity) for any RDT, the reactivity of that batch of RDT was verified using a positive control sample (frozen at − 80 °C).

### Data analysis

Stability was defined as the number of weeks the controls or the stabilized sample remained positive as shown by their reactivity on RDTs. The limit of stabilization of a chemical additive is the duration in which the stabilized sample (both culture and patient sample) tested positive for all the three RDTs. CareStart RDT was not included in the analysis because sample retesting could not be conducted to week 53 of storage; due to lack of similar lot number of the kit in the market. Percentage stability of a sample was calculated against week 53, the endpoint [No. of weeks sample was reactive/53 weeks × 100%]. Average percentage stability was computed by combining both the culture and patient samples percentage stabilities. The Z-test for comparing proportions [27] was used to compare the percentage stability of the stabilized sample proteins against the non-stabilized sample proteins. To determine the percentage increase in stability of chemically stabilized HRP2, PLDH, and aldolase, [the time taken for a stabilized antigen (in weeks) to lose reactivity on the RDTs was compared to the time taken for non-stabilized antigen to lose reactivity on the RDTs]. The mean percentage increase in stability for each chemical additive was computed from three RDT results for HRP2 or PAN antigens at each parasite density.

## Results

### Stability of HRP2, pLDH, and aldolase in non-stabilized dried *P. falciparum*-infected samples

To determine the temporal stability of the RDT target *Plasmodium* proteins, we measured reactivity of the non-stabilized samples for 53 weeks of storage using three malaria kits. HRP2 from culture and patient samples retained stability for 24 weeks at room temperature as indicated by its reactivity on SD Bioline, First Response, and BinaxNOW malaria RDTs. But, the stability was slightly different (39.6%) in patient samples when measured with First Response at 2000 and 500 P/µL, and BinaxNOW at 500 P/µL (Table 3).

**Table 3:** Baseline stability of *Plasmodium* HRP2, pLDH, and aldolase in non-stabilized samples (study positive controls) during the 53 weeks of analysis

Sample type	Parasite concentration	Malaria RDTs	HRP 2		pLDH and aldolase	
			Reactivity (in weeks)	Percentage stability	Reactivity (in weeks)	Percentage stability
Culture Sample	2000 P/µL	SD Bioline	24	45.28	12	22.64
		First Response	24	45.28	12	22.64
		BinaxNOW	24	45.28	18	33.96
	500 P/µL	SD Bioline	24	45.28	6	11.32
		First Response	24	45.28	6	11.32
		BinaxNOW	24	45.28	2	3.77
	200 P/µL	SD Bioline	24	45.28	0	0.00
		First Response	24	45.28	0	0.00
		BinaxNOW	24	45.28	0	0.00
Patient Sample	2000 P/µL	SD Bioline	24	45.28	12	22.64
		First Response	21	39.62	12	22.64
		BinaxNOW	24	45.28	2	3.77
	500 P/µL	SD Bioline	24	45.28	4	7.55
		First Response	21	39.62	2	3.77
		BinaxNOW	21	39.62	2	3.77
	200 P/µL	SD Bioline	24	45.28	0	0.00
		First Response	24	45.28	0	0.00
		BinaxNOW	24	45.28	0	0.00

P/µL, Parasites per microliter; RDTs, rapid diagnostic Tests; pLDH, *Plasmodium* lactate dehydrogenase; HRP2, histidine rich protein II

*Plasmodium* LDH in cultured samples was shown to lose stability in less than 12 weeks. In patient samples, reactivity of pLDH was retained for 12 weeks at 2000 P/µL when evaluated using SD Bioline and First Response. But, stability was shown to reduce as parasite concentration reduced in both patient and culture samples as shown by SD Bioline at 2000 P/µL (22.64%) to 11.32% at 500 P/µL (Table 3).

*Plasmodium* aldolase was shown to retain stability for 18 weeks of storage at 2000 P/µL as measured by BinaxNOW but reduced to 2 weeks at 500 P/µL in culture samples. The patient samples showed reactivity for aldolase for only 2 weeks of storage because reactivity was not measured for several time points due to lack of similar lot number kits. At 200 P/µL, the study reports that PAN antigens were not reactive from baseline analysis in both the culture and patient samples (Table 3).

### Stability of HRP2, pLDH, and aldolase present in stabilized dried *P. falciparum*-infected samples

To determine the effect of chemical additives on the temporal stability of RDT target antigens, we measured reactivity of the stabilized samples for 53 weeks of storage using four RDTs. The limit of stabilization for each chemical additive was shown vary across parasite density levels. Biostab enzyme stabilizer combined with sucrose or trehalose had the best limit of stabilization for 53 weeks across all the parasite densities for HRP2 although their limit of stabilization was lower for PAN antigens (2–24 weeks). Sucrose combined with trehalose had the best limit of stabilization (53 weeks) for PAN antigens at 2000 p/µL but the limit of stabilization was lower (18 weeks) at 500 p/µL (Table 4).

**Table 4:** Limits of stabilization (in weeks) for each chemical additive at different parasite densities; on improving the stability of HRP2, pLDH and aldolase present in dried *P. falciparum*-infected samples

Chemical additives	HRP2						pLDH and aldolase			
	2000 P/µL		500 P/µL		200 P/µL		2000 P/µL		500 P/µL	
	Stability (in weeks)	Percentage stability (%)	Stability (in weeks)	Percentage stability (%)	Stability (in weeks)	Percentage stability (%)	Stability (in weeks)	Percentage stability (%)	Stability (in weeks)	Percentage stability (%)
Sucrose	53	100	53	100	33	62	21	40	0	0
Glycerol/sucrose	33	62	33	62	21	40	18	34	18	34
Alsever’s/sucrose	53	100	53	100	53	100	12	23	2	4
Biostab/sucrose	53	100	53	100	53	100	2	4	2	4
Trehalose	53	100	53	100	43	81	43	81	21	40
Sucrose/trehalose	53	100	43	81	33	62	53	100	18	34
Glycerol/trehalose	53	100	24	45	24	45	12	23	10	19
Biostab/trehalose	53	100	53	100	53	100	24	45	2	4
LDH stabilizer	53	100	53	100	33	62	15	28	2	4
LDH stabilizer/trehalose	43	81	43	81	33	62	24	45	10	19

P/µL, Parasites per microliter; pLDH, *Plasmodium* lactate dehydrogenase; HRP2, histidine rich protein II

### Sucrose

**Fig. 1:**
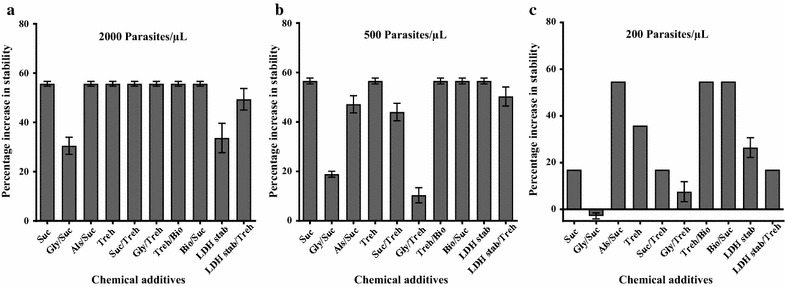
Mean percentage increase in stability of HRP2 antigen in stabilized samples (chemical additive added) compared to the control samples (no chemical additive added). Error bars: indicate mean and standard error of the mean. **a** The percentage increase in stability of pLDH and aldolase in stabilized samples at 2000 p/µL. **b** Indicates stabilized samples at 500 p/µL. **c** Shows stabilized samples at 200 p/µL. *Suc* sucrose, *Gly/Suc* glycerol combined with sucrose, *Als/Suc* Alsever’s solution combined with sucrose, *Treh* trehalose, *Suc/Treh* sucrose combined with trehalose, *Gly/Treh* glycerol combined with trehalose, *Treh/Bio* trehalose combined with biostab enzyme stabilizer, *Bio/Suc* biostab enzyme stabilizer combined with sucrose, *LDH stab* lactose dehydrogenase stabilizer, *LDH stab/Treh* lactose dehydrogenase stabilizer combined with trehalose

Histidine rich protein II stabilized by sucrose showed 100% stability at 500 and 2000 p/µL, but stability reduced to 62% at 200 p/µL on all the three RDTs. Combining sucrose with Alsever’s solution or biostab enzyme stabilizer enhanced stability of HRP2 at 200 p/µL up to 100% during the storage period. Combining glycerol with sucrose decreased stability to 81, 62 and 40% at 2000, 500 and 200 p/µL respectively (Additional file 1: Table S1). The percentage increase in stability of HRP2 was evaluated in the presence of sucrose and its combinations when compared to the non-stabilized samples. Sucrose alone and its combinations with Alsever’s solution or biostab stabilizer increased the stability of HRP2 by an average of 56% (p < 0.0001), while addition of glycerol increased the stability of HRP2 slightly by 30% (p < 0.05) at 2000 p/µL) (Fig. 1).

**Fig. 2:**
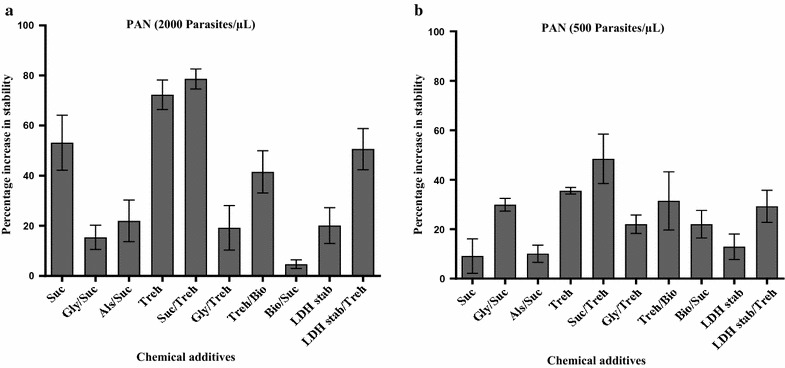
Mean percentage increase in stability of PAN antigens in stabilized samples (chemical additive added) compared to the control samples (no chemical additive added). Error bars: indicate mean and standard error of the mean. **a** The percentage increase in stability of pLDH and aldolase in stabilized samples at 2000 p/µL, while **b** indicates stabilized samples at 500 p/µL. *Suc* sucrose, *Gly/Suc* glycerol combined with sucrose, *Als/Suc* Alsever’s solution combined with sucrose, *Treh* trehalose, *Suc/Treh* sucrose combined with trehalose, *Gly/Treh* glycerol combined with trehalose, *Treh/Bio* trehalose combined with biostab enzyme stabilizer, *Bio/Suc* biostab enzyme stabilizer combined with sucrose, *LDH stab* lactose dehydrogenase stabilizer, *LDH stab/Treh* lactose dehydrogenase stabilizer combined with trehalose

Sucrose stabilized PAN antigens in culture samples by 100% at 2000 p/µL, but stability was lower at 81% in patient samples when measured by SD Bioline and BinaxNOW. Unlike HRP2, combination with Alsever’s solution, biostab enzyme stabilizer, or glycerol decreased stability of PAN antigens at 2000 p/µL compared to sucrose alone (Additional file 1: Table S2). There was a significant percentage increase (53%) in stability of PAN antigens in the presence of sucrose alone than in combination with Alsever’s solution (22%, p < 0.001). Glycerol or biostab stabilizer decreased the stabilizing ability of sucrose significantly on pLDH and aldolase at 2000 p/µL, but these combinations increased the stability of the PAN antigens significantly at (500 p/µL) by 29 and 22% respectively (p < 0.001) (Fig. 2). These results indicated that sucrose alone or its combinations had variable impacts on the stability of HRP2, pLDH and aldolase.

### Trehalose

Trehalose alone stabilized HRP2 for the whole storage duration (53 weeks) in concentrations of 2000 and 500 p/µL but only 43 weeks for 200 p/µL. In combination with biostab enzyme stabilizer, the HRP2 retained reactivity on all RDTs (100% stability) at all three parasite concentrations. When trehalose was combined with sucrose, there was 100% stability at 2000 p/µL, but stability reduced to 45–81% at 500 p/µL and 45–62% at 200 p/µL. A combination with glycerol reduced the stability further to 40–62% at lower concentrations despite retaining 100% stability at 2000 p/µL (Additional file 1: Table S3). Compared to the control samples, the average percentage increase in stability of HRP2 was 56% at 2000 p/µL in the presence of trehalose and its combinations (p < 0.0001). The percentage increase in stability of HRP2 was significant in the presence of trehalose and in combination with biostab enzyme stabilizer at both 500 and 200 p/µL (p < 0.001) (Fig. 1).

In the presence of trehalose alone the PAN antigens retained reactivity for 43–53 weeks of storage at 2000 p/µL, but reactivity was reduced to 40–45% at 500 p/µL. The combination of trehalose and glycerol had a stability of 23–62% at 2000 p/µL and 19–34% at 500 p/µL, while the combination with biostab enzyme stabilizer had slightly higher stability of 45–81% at 2000 p/µL but was highly variable at 500 p/µL (4–62%). (Additional file 1: Table S4). There was a significant percentage increase in stability of PAN antigens by 72% in trehalose alone, 78% in combination with sucrose, 42% in combination with biostab enzyme stabilizer, and 19% in combination with glycerol at 2000 p/µL (p < 0.0001) (Fig. 2).

Trehalose alone and its combination with biostab enzyme stabilizer improved the stability of both HRP2 and PAN antigens consistently throughout the study duration. Of note is that trehalose combination with biostab enzyme stabilizer provided the best chemical additive to improve stability of the dried *P. falciparum*-infected samples due to the ease of reconstitution as compared to trehalose alone.

### LDH stabilizer

Lactate dehydrogenase stabilizer improved the stability ofHRP2 during the storage although there was variability in detection by the three RDTs with stability ranging from 62% BinaxNOW to 100% for SD Bioline at 2000 p/µL. But the study shows that at lower concentration (500 p/µL), reactivity was retained for the study duration but slightly decreased to 62–81% at 200 p/µL. In combination with trehalose, stability was 81–100% at both 2000 and 500 p/µL and 62% at 200 p/µL. (Additional file 1: Table S5). This represents a significant percentage increase in stability when compared to control samples (p < 0.001) (Fig. 1).

LDH stabilizer also maintained the stability of pLDH for 12–33 weeks at 2000 p/µL, but reduced to 10–21 weeks at 500 p/µL. The stabilizing ability of LDH stabilizer on aldolase was shown to be minimized from 24 weeks at 2000 p/µL to 2 weeks at 500 p/µL. (Additional file 1: Table S6). Addition of trehalose improved the stability of aldolase to 21 and 24 weeks in culture and patient sample respectively. This corresponds to significant increase in average percentage stability by 50% at 2000 p/µL and 29% at 2000 p/µL (p < 0.01) (Fig. 2).

## Discussion

This study indicates that chemical additives significantly improve the long-term storage stability of HRP2, pLDH, and aldolase as determined by reactivity on the three malaria RDTs (SD Bioline, First Response, and BinaxNOW). Stabilization of dried *P. falciparum*-infected samples by sucrose, trehalose, biostab/trehalose, LDH stabilizer/trehalose, and trehalose/sucrose in turn improved the stability of all the three proteins during the 1 year of storage. Previous studies have shown that through preferential interactions these chemical additives improve the long-term stability of various proteins including; ribonuclease A, lysozyme, chymo-trypsinogen recombinant interleukin-1 receptor, monoclonal antibodies, pyro-phosphatase, bovine serum albumin, ribosomal protein S6, cutinase, LDH and lysozyme [12, 15, 20, 24, 25, 28–30]. The findings of the present study show that stabilization of the target proteins ensures prolonged storage of dried *Plasmodium falciparum*-infected samples in ambient temperature conditions and can be used as positive controls for validation of malaria RDTs at the point of care.

The results on percentage increase in stability clearly indicate that the parasite density was not a factor in conferring stability to the proteins, but loss of reactivity was probably due low levels of the antigens in the samples. As observed, stabilization of the target proteins at 200 p/µL did not prevent their loss of reactivity quicker (1 + to 0) than samples at 2000 p/µL (4 + to 0). Consistent with this findings, Aidoo et al. documented the loss of reactivity in the samples standardized at 200 p/µL as shown by ten different malaria kits was quicker as compared to samples at 2000 p/µL [9]. The present study shows that *Plasmodium* LDH and aldolase were detectable at baseline by all the four RDTs, but the reactivity of antigens declined quickly with decrease in parasite density during storage. A study by Versteg and Mens indicated that none of the samples containing a parasite density of 300 p/μL gave a signal throughout their study while the samples containing 3000 p/μL were positive for 4 weeks and samples at 30,000 p/μL yielded a signal that remained visible for some time [31].

These results indicate that stability of HRP2, LDH and aldolase increased as concentration of the chemical additives decreased. The percentage temporal stability of all the proteins at 2000 p/µL was lower than 500 p/µL and it increased further at 200 p/µL on SD Bioline, First Response, and BinaxNOW malaria. This can be due to the dilution effect and surface area to volume ratio [32] whereby there is a specific distribution of the additives around the proteins at a particular concentration and size of the protein. In theory, if the protein concentration is reduced and the same amount of the additive is maintained the proteins achieve more stability because all the binding sites of the protein will be saturated depending on the molecular weight. A previous study on effect of sucrose concentration on protein stability showed that; a 1:1 weight ratio provided a sixfold stabilization toward aggregation for hGH (22 kD), a fourfold stabilization for rHSA (66 kD), and a 20-fold stabilization for IgG1 antibody (150 kD) protein [32].

Glycerol/trehalose and glycerol/sucrose are the only additives shown to destabilize or decrease stability of HRP2, LDH, or aldolase. A previous study has shown that glycerol is also preferentially excluded from the surface of the protein, but the exclusion by glycerol is thermodynamically unsuitable, as it favours the unstructured form of proteins [33].

The main challenges observed in this study include; differences in the stabilization of HRP2 antigen versus the PLDH and aldolase antigen, low parasite concentration affecting possible outcomes, and noticeable dissimilarity in test performances between the three malaria kits. The differences between stabilization of the proteins can be due levels of the antigens in the sample. This is because different patients have varying expression profiles of the parasite antigens [34] and LDH/aldolase levels can be very low in patient sample as compared to HRP2. Martin et al. showed that the levels of HRP2 are higher in individuals (approximately sevenfold), and can persist in the bloodstream for more than 2 weeks as compared to the PLDH which cannot be detected in smaller volumes of blood and the clearance time rate is less than 5 days [35].

The noticeable dissimilarity between different RDTs can be due to manufacturer differences, and lot to lot or batch to batch variation. Previous studies on patient samples and WHO panel detection scores have shown that the performance of malaria RDTs varies between different manufacturer brands and between different lots from the same manufacturer [2, 3, 6, 7, 9]. Manufacturer factors, such as the type of immunoglobulin used for antigen capture, the type of strip used, type of buffer used, the quality of fluorescence particles, and the overall kit development, play a critical role in the variability of results among kits [7]. In field use (health facilities), the RDTs have several limitations, such as decreased and variable sensitivity at lower parasite densities, false negative results, and false positive results [3, 36].

## Conclusion

This study demonstrated that the presence of chemical additives in dried *P. falciparum*-infected samples significantly improves the long-term stability (~ 53 weeks) of HRP2, PLDH, and aldolase. Specifically, sucrose, trehalose, sucrose/trehalose, biostab/trehalose demonstrated the ability to significantly improve the stability of the RDT target antigens. This study also recommends that future exploration in the field be carried out on the use of biostab/trehalose stabilized dried *P. falciparum*-infected samples. Stabilized dried *P. falciparum*-infected samples as RDT positive controls should be sent to off-site facilities in different climatic zones both in Kenya and other countries for testing under ambient temperature conditions in-order to determine their field operational viability.

## Additional file

**Additional file 1.** Percentage stability of *Plasmodium* HRP2, pLDH, and aldolase after 53 weeks of storage tested on three malaria RDTs after stabilization using chemical additives.
