# Supplementary material for: Stabilization of RDT target antigens present in dried Plasmodium falciparum-infected samples for validating malaria rapid diagnostic tests at the point of care
Source: Malar J. 2018 Jan 8;17:10. doi: 10.1186/s12936-017-2155-7 (PMC5759799; doi:10.1186/s12936-017-2155-7)
Supplement: Supplementary file 1 — Additional file 1. Percentage stability of Plasmodium HRP2, pLDH, and aldolase after 53 weeks of storage tested on three malaria RDTs after stabilization using chemical additives. [file 12936_2017_2155_MOESM1_ESM.docx]

**Additional file 1 Percentage stability of *Plasmodium* HRP2, pLDH, and aldolase after 53 weeks of storage tested on three malaria RDTs after stabilization using chemical additives**

**Table S1 Sucrose and its combinations on HRP2 antigen**

|  |  | **2000P/µL** | | **500P/µL** | | **200P/µL** | |
| --- | --- | --- | --- | --- | --- | --- | --- |
|  |  | **Culture** | **Patient** | **Cultures** | **Patient** | **Cultures** | **Patient** |
| **Additives** | **RDTs** | **WK *(St)*** | **WK *(St)*** | **WK *(St)*** | **WK *(St)*** | **WK *(St)*** | **WK *(St)*** |
| **Sucrose** | SD Bioline | 53 (100%) | 53 (100%) | 53 (100%) | 53 (100%) | 33 (62%) | 33 (62%) |
|  | First Response | 53 (100%) | 53 (100%) | 53 (100%) | 53 (100%) | 33 (62%) | 33 (62%) |
|  | BinaxNOW | 53 (100%) | 53 (100%) | 53 (100%) | 53 (100%) | 33 (62%) | 33 (62%) |
| **Glycerol/Sucrose** | SD Bioline | 43 (81%) | 43 (81%) | 33 (62%) | 33 (62%) | 21 (40%) | 21 (40%) |
|  | First Response | 33 (62%) | 33 (62%) | 33 (62%) | 33 (62%) | 24 (45%) | 24 (45%) |
|  | BinaxNOW | 43 (81%) | 43 (81%) | 33 (62%) | 33 (62%) | 24 (45%) | 21 (40%) |
| **Alsever’s/Sucrose** | SD Bioline | 53 (100%) | 53 (100%) | 53 (100%) | 43 (81%) | 53 (100%) | 53 (100%) |
|  | First Response | 53 (100%) | 53 (100%) | 53 (100%) | 43 (81%) | 53 (100%) | 53 (100%) |
|  | BinaxNOW | 53 (100%) | 53 (100%) | 53 (100%) | 43 (81%) | 53 (100%) | 53 (100%) |
| **Biostab/Sucrose** | SD Bioline | 53 (100%) | 53 (100%) | 53 (100%) | 53 (100%) | 53 (100%) | 53 (100%) |
|  | First Response | 53 (100%) | 53 (100%) | 53 (100%) | 53 (100%) | 53 (100%) | 53 (100%) |
|  | BinaxNOW | 53 (100%) | 53 (100%) | 53 (100%) | 53 (100%) | 53 (100%) | 53 (100%) |

P/µL-Parasites per microlitre, WK – Weeks stable, St– Percentage stability, RDTs –Rapid diagnostic Tests, Patient-Patient samples, Culture- Culture samples

**Table S2 Sucrose and its combinations on PLDH and aldolase antigens**

|  |  | **2000P/µL** | | **500P/µL** | |
| --- | --- | --- | --- | --- | --- |
|  |  | **Culture** | **Patient** | **Cultures** | **Patient** |
| **Additives** | **RDTs** | **WK *(St)*** | **WK *(St)*** | **WK *(St)*** | **WK *(St)*** |
| **Sucrose** | SD Bioline | 53 (100%) | 43 (81%) | 15 (28%) | 0 (0%) |
|  | First Response | 21 (40%) | 24 (45%) | 15 (28%) | 0 (0%) |
|  | BinaxNOW | 53 (100%) | 43 (81%) | 21 (40%) | 0 (0%) |
| **Glycerol/Sucrose** | SD Bioline | 18 (34%) | 21 (40%) | 18 (34%) | 24 (45%) |
|  | First Response | 18 (34%) | 21 (40%) | 18 (34%) | 18 (34%) |
|  | BinaxNOW | 18 (34%) | 21 (40%) | 18 (34%) | 21 (40%) |
| **Alsever’s/Sucrose** | SD Bioline | 24 (45%) | 18 (34%) | 15 (28%) | 15 (28%) |
|  | First Response | 18 (34%) | 12 (23%) | 12 (23%) | 8 (15%) |
|  | BinaxNOW | 33 (62%) | 33 (62%) | 2 (4%) | 2 (4%) |
| **Biostab/Sucrose** | SD Bioline | 18 (34%) | 15 (28%) | 21 (40%) | 15 (28%) |
|  | First Response | 15 (28%) | 12 (23%) | 15 (28%) | 15 (28%) |
|  | BinaxNOW | 21 (40%) | 2 (4%) | 2 (4%) | 24 (45%) |

P/µL-Parasites per microlitre, WK – Weeks stable, St– Percentage stability, RDTs –Rapid diagnostic Tests, Patient-Patient samples, Culture- Culture samples

**Table S3 Trehalose and its combinations on HRP2 antigen**

|  |  | **2000P/µL** | | **500P/µL** | | **200P/µL** | |
| --- | --- | --- | --- | --- | --- | --- | --- |
|  | **RDTs** | **Culture** | **Patient** | **Cultures** | **Patient** | **Cultures** | **Patient** |
| **Additives** |  | **WK *(St)*** | **WK *(St)*** | **WK *(St)*** | **WK *(St)*** | **WK *(St)*** | **WK *(St)*** |
| **Trehalose** | SD Bioline | 53 (100%) | 53 (100%) | 53 (100%) | 53 (100%) | 43 (81%) | 43 (81%) |
|  | First Response | 53 (100%) | 53 (100%) | 53 (100%) | 53 (100%) | 43 (81%) | 43 (81%) |
|  | BinaxNOW | 53 (100%) | 53 (100%) | 53 (100%) | 53 (100%) | 43 (81%) | 43 (81%) |
| **Sucrose/Trehalose** | SD Bioline | 53 (100%) | 53 (100%) | 53 (100%) | 53 (100%) | 33 (62%) | 33 (62%) |
|  | First Response | 53 (100%) | 53 (100%) | 43 (81%) | 43 (81%) | 33 (62%) | 33 (62%) |
|  | BinaxNOW | 53 (100%) | 53 (100%) | 43 (81%) | 43 (81%) | 33 (62%) | 33 (62%) |
| **Glycerol/Trehalose** | SD Bioline | 53 (100%) | 53 (100%) | 33 (62%) | 24 (45%) | 24 (45%) | 33 (62%) |
|  | First Response | 53 (100%) | 53 (100%) | 33 (62%) | 24 (45%) | 21 (40%) | 33 (62%) |
|  | BinaxNOW | 53 (100%) | 53 (100%) | 33 (62%) | 24 (45%) | 24 (45%) | 33 (62%) |
| **Biostab/Trehalose** | SD Bioline | 53 (100%) | 53 (100%) | 53 (100%) | 53 (100%) | 53 (100%) | 53 (100%) |
|  | First Response | 53 (100%) | 53 (100%) | 53 (100%) | 53 (100%) | 53 (100%) | 53 (100%) |
|  | BinaxNOW | 53 (100%) | 53 (100%) | 53 (100%) | 53 (100%) | 53 (100%) | 53 (100%) |

P/µL-Parasites per microlitre, WK – Weeks stable, St– Percentage stability, RDTs –Rapid diagnostic Tests, Patient-Patient samples, Culture- Culture samples

**Table S4 Trehalose and its combinations on PLDH and Aldolase antigens**

|  |  | **2000P/µL** | | **500P/µL** | |
| --- | --- | --- | --- | --- | --- |
|  |  | **Culture** | **Patient** | **Cultures** | **Patient** |
| **Additives** | **RDTs** | **WK *(St)*** | **WK *(St)*** | **WK *(St)*** | **WK *(St)*** |
| **Trehalose** | SD Bioline | 53 (100%) | 53 (100%) | 24 (45%) | 21 (40%) |
|  | First Response | 43 (81%) | 43 (81%) | 24 (45%) | 21 (40%) |
|  | BinaxNOW | 53 (100%) | 53 (100%) | 24 (45%) | 21 (40%) |
| **Sucrose/Trehalose** | SD Bioline | 53 (100%) | 53 (100%) | 33 (62%) | 15 (28%) |
|  | First Response | 53 (100%) | 53 (100%) | 24 (45%) | 18 (34%) |
|  | BinaxNOW | 53 (100%) | 53 (100%) | 43 (81%) | 43 (81%) |
| **Glycerol/Trehalose** | SD Bioline | 24 (45%) | 24 (45%) | 18 (34%) | 18 (34%) |
|  | First Response | 12 (23%) | 12 (23%) | 10 (19%) | 10 (19%) |
|  | BinaxNOW | 24 (45%) | 33 (62%) | 18 (34%) | 18 (34%) |
| **Biostab/Trehalose** | SD Bioline | 43 (81%) | 43 (81%) | 33 (62%) | 12 (23%) |
|  | First Response | 24 (45%) | 33 (62%) | 24 (45%) | 8 (15%) |
|  | BinaxNOW | 24 (45%) | 33 (62%) | 43 (81%) | 2 (4%) |

P/µL-Parasites per microlitre, WK – Weeks stable, St– Percentage stability, RDTs –Rapid diagnostic Tests, Patient-Patient samples, Culture- Culture samples

**Table S5 Lactose Dehydrogenase (LDH) stabilizer and it combination on HRP2 antigen**

|  |  | **2000P/µL** | | **500P/µL** | | **200P/µL** | |
| --- | --- | --- | --- | --- | --- | --- | --- |
|  |  | **Culture** | **Patient** | **Cultures** | **Patient** | **Cultures** | **Patient** |
| **Additives** | **RDTs** | **WK *(St)*** | **WK *(St)*** | **WK *(St)*** | **WK *(St)*** | **WK *(St)*** | **WK *(St)*** |
| **LDH stabilizer** | SD Bioline | 43 (100%) | 43 (81%) | 53 (100%) | 53 (100%) | 33 (62%) | 43 (81%) |
|  | First Response | 43 (81%) | 43 (81%) | 53 (100%) | 53 (100%) | 33 (62%) | 43 (81%) |
|  | BinaxNOW | 33 (62%) | 33 (62%) | 53 (100%) | 53 (100%) | 33 (62%) | 43 (81%) |
| **LDH stabilizer/** | SD Bioline | 53 (100%) | 53 (100%) | 53 (100%) | 53 (100%) | 33 (62%) | 33 (62%) |
| **Trehalose** | First Response | 53 (100%) | 53 (100%) | 43 (81%) | 43 (81%) | 33 (62%) | 33 (62%) |
|  | BinaxNOW | 43 (81%) | 43 (81%) | 53 (100%) | 53 (100%) | 33 (62%) | 33 (62%) |

P/µL-Parasites per microlitre, WK – Weeks stable, St– Percentage stability, RDTs –Rapid diagnostic Tests, Patient-Patient samples, Culture- Culture samples

**Table S6 Lactose Dehydrogenase stabilizer (LDH) and it combination PLDH and Aldolase antigens**

|  |  | **2000P/µL** | | **500P/µL** | |
| --- | --- | --- | --- | --- | --- |
|  |  | **Culture** | **Patient** | **Cultures** | **Patient** |
| **Additives** | **RDTs** | **WK (St)** | **WK (St)** | **WK (St)** | **WK (St)** |
| **LDH stabilizer** | SD Bioline | 33 (62%) | 24 (45%) | 21 (40%) | 18 (34%) |
|  | First Response | 15 (28%) | 12 (23%) | 10 (19%) | 10 (19%) |
|  | BinaxNOW | 24 (45%) | 24 (45%) | 2 (4%) | 2 (4%) |
| **LDH stabilizer/Trehalose** | SD Bioline | 43 (81%) | 33 (62%) | 33 (62%) | 15 (28%) |
|  | First Response | 33 (62%) | 24 (45%) | 12 (23%) | 10 (19%) |
|  | BinaxNOW | 53 (100%) | 43 (81%) | 21 (40%) | 24 (45%) |

P/µL-Parasites per microlitre, WK – Weeks stable, St– Percentage stability, RDTs –Rapid diagnostic Tests, Patient-Patient samples, Culture- Culture samples
